# Healthcare Waste Management: connections with sustainable nursing care

**DOI:** 10.1590/1980-220X-REEUSP-2023-0229en

**Published:** 2024-10-28

**Authors:** Maria José Carvalho Ferreira, Carla Aparecida Arena Ventura, Glaucia Valente Valadares, Isabel Amélia Costa Mendes, Thiago Privado da Silva, Ítalo Rodolfo Silva

**Affiliations:** 1 Universidade Federal do Rio de Janeiro, Escola de Enfermagem Anna Nery, Rio de Janeiro, RJ, Brazil.; 2 Universidade de São Paulo, Escola de Enfermagem de Ribeirão Preto, Ribeirão Preto, SP, Brazil.

**Keywords:** Medical Waste, Nursing Service, Hospital, Nursing, Sustainable Development, Residuos Sanitarios, Servicio de Enfermería en Hospital, Enfermería, Desarrollo Sostenible, Resíduos de Serviços de Saúde, Serviço Hospitalar de Enfermagem, Enfermagem, Desenvolvimento Sustentável

## Abstract

**Objective:**

To understand the meanings revealed by nurses about sustainable care in its connections with the management of Healthcare Waste in hospital.

**Method:**

Qualitative research, whose theoretical and methodological references were, respectively, Complexity Theory and Grounded Theory. Nursing professionals from a public hospital in Rio de Janeiro participated in the study. Data was collected through semi-structured interviews.

**Results:**

The actions-interactions signified by the nursing staff, for the management of Healthcare Waste, revealed an understanding of the socio-environmental responsibility of the rational use of material resources; of the need for permanent education to promote environmental education; of the need to supervise the proper management of waste. The professionals perceive themselves as a driving force for reordering positive changes in this context.

**Conclusion:**

The participants signaled implications between knowledge, rational use of materials, waste generation and disposal, health economics and quality of care. From these connections, we can deduce the complex meaning of sustainable hospital nursing care in the context of Healthcare Waste management.

## RESUMO

**Objetivo:**

Compreender os significados desvelados pela enfermagem sobre o cuidado sustentável em suas conexões com o gerenciamento de Resíduos de Serviços de Saúde no hospital.

**Método:**

Pesquisa qualitativa, cujos referenciais teórico e metodológico foram, respectivamente, a Teoria da Complexidade e a Teoria Fundamentada nos Dados. Participaram do estudo profissionais de enfermagem de um hospital público do Rio de Janeiro. Os dados foram coletados por entrevistas semiestruturadas.

**Resultados:**

As ações-interações significadas pela enfermagem, para o gerenciamento dos Resíduos dos Serviços de Saúde, revelaram a compreensão sobre a responsabilidade socioambiental do uso racional de recursos materiais; da necessidade de educação permanente para promover educação ambiental; da necessidade de supervisão o gerenciamento adequado dos resíduos. Os profissionais se percebem como força motriz para reordenar mudanças positivas nesse contexto.

**Conclusão:**

Os participantes sinalizaram implicações entre conhecimento, uso racional de materiais, geração e descarte de resíduos, economia da saúde e qualidade da assistência. Dessas conexões, depreende-se o sentido complexo de cuidado sustentável da enfermagem hospitalar no contexto do gerenciamento de Resíduos dos Serviços de Saúde.

## RESUMEN

**Objetivo:**

Comprender los significados revelados por enfermeros sobre cuidados sostenibles en sus conexiones con la Gestión de Residuos Sanitarios en el hospital.

**Método:**

Investigación cualitativa, cuyos referentes teóricos y metodológicos fueron, respectivamente, la Teoría de la Complejidad y la Teoría Fundamentada en Datos. Participaron en el estudio profesionales de enfermería de un hospital público de Río de Janeiro. Los datos se recogieron mediante entrevistas semiestructuradas.

**Resultados:**

Las acciones-interacciones significadas por el personal de enfermería, para la gestión de Residuos de Servicios de Salud, revelaron la comprensión de la responsabilidad socioambiental del uso racional de los recursos materiales; de la necesidad de educación permanente para promover la educación ambiental; de la necesidad de supervisar la correcta gestión de los residuos. Los profesionales se perciben a sí mismos como un motor para reordenar cambios positivos en este contexto.

**Conclusión:**

Los participantes señalaron implicaciones entre el conocimiento, el uso racional de materiales, la generación y eliminación de residuos, la economía de la salud y la calidad de la atención. De estas conexiones se deduce el complejo significado de los cuidados sostenibles de enfermería hospitalaria en el contexto de la gestión de residuos de los servicios de salud.

## INTRODUCTION

Those environments that provide human healthcare and generate hazardous waste are considered to be originators of Healthcare Waste (HCW), namely: those with risk potential due to the presence of biological materials capable of causing infection; sharp objects; hazardous chemicals and radioactive materials^(1)^. In this way, the challenges imbued in the context of HCW are relevant to global and local discussions due to the significant concern that public bodies and private sectors should be expected to have with the preservation of natural resources, as well as public health^(2)^.

The actions of healthcare, which include those carried out in a hospital environment, various materials are used, and as a consequence of this reality, different types of waste are generated. When managed improperly, this waste can pose risks to people’s health and the environment^(2,3)^. These impacts have a significant impact on minority populations and the poorest people, who are exposed to the risks posed by the lack of adequate sanitary conditions when compared to more affluent populations^(2)^. Therefore, the incorporation of environmental practices and values in all social spheres should be a matter of concern for the health professions, which particularly involves nursing, not only because it is the profession with the largest contingent of human resources in the health sector, but also because of the quality and projection of its work. From this reality, there are possibilities to better promote health practices that are more sustainable and healthier for the environment, people and communities^(4)^.

As with any complex phenomenon, in addition to its local interactions, seen from the point of view of the work carried out by nursing professionals, we must also consider its planetary projection in terms of its impacts and origins^(5)^, including the use of safer and more sustainable practices in the management of HCW. This includes encouraging the use of greener products and technologies and promoting environmental awareness and education among patients and their families^(6)^.

Thus, from a complex perspective, using the hologrammatic principle^(5)^ in which the whole is in the part and the part in the whole, it can be understood that sustainability must be thought of in a logic that is not limited to the environment, but involves the individuals and groups that interact with it. Therefore, working on sustainability should be the prerogative of everyone involved in the healthcare process^(5,7)^.

Sustainability is commonly conceptualized based on a dialogue between the definitions of ecology and holism. However, for complexity thinking seen from the perspective of Morin^(5)^, the idea of holism should be rejected when it does not value the identity of the parts. In this context, sustainability, from a hologrammatic perspective, must be recognized in its connections between health, culture, the environment and society, whose interactions between each part affect the surrounding environment^(5,8)^.

For this problematization, it is important to mention that Complexity Theory^(5)^ conceives risks, illusions and uncertainties as inseparable elements of human relations. Furthermore, complex thinking considers that in order to deal with these elements it is necessary to develop strategies. This understanding seems to corroborate the concept of sustainability from the perspective of ecology, which evaluates responses to environmental dangers to the health, quality and safety of the physical environment. Other authors, however, suggest that an ecological perspective in human society can only be developed through balance in nature, with a focus on the immediate environment and the relevance of the global situation^(8,9)^.

Whether in local or global projections, nursing initiatives are remarkable throughout history for been applied towards sustainability, because nurses strive to improve human health in the physical, economic and social environments. Because of its complex perspective, nursing is positioned to affect the present and future of sustainability^(8,10)^, based on care that values the parts that make up the whole/environment in order to better deal with natural resources^(8)^.

Under the perspective of complexity, the meaning of complex is to recognize the multidimensionality of phenomena, therefore this research assumes the epistemological and ontological perspective that nursing care seeks sustainability by valuing the environment in its metaparadigm, and thus it has the potential for environmental awareness. In addition, environmental awareness is an important factor that directly influences HCW management and can be a key factor in promoting environmental sustainability, as well as contributing to disease prevention and public health promotion. Therefore, it can be seen that nursing care is complex when it involves, in its multidimensionality, sustainability itself.

The complexity that stems from ecological awareness to strategic actions means that nursing can, through public policies, contemplate broad conjunctures to achieve global agendas, such as the UN’s 2030 Agenda, to achieve the 17 Sustainable Development Goals (SDGs), including those that deal, directly or indirectly, with the substantial reduction of waste generation through prevention, reduction, recycling and reuse; links between health, well-being, sustainable consumption and production; action against global climate change, sustainable cities and communities, among others^(11)^.

In view of the above, the problematic of this research focuses on the premise that ecological awareness starts from the deepest field of interpretation of reality when such awareness reaches the field of meanings, and these, therefore, mobilize human/social actions/behaviors. In this sense, it is important to ask: what actions and interactions permeate the meanings revealed by nursing professionals, in the hospital context, in the midst of the care aimed at sustainability involved in the management of HCW?

The aim of this research was therefore, to understand the meanings revealed by nursing staff about sustainable care in its connections with the management of Healthcare Waste in the hospital.

## METHOD

### Study Design

A qualitative study based on the theoretical framework of the Theory of Complexity, from Edgar Morin’s perspective^(5)^. For the analytical process, we used Grounded Theory (GT), from the Corbinian school^(12,13)^. This method consists of an intense comparative analysis between stages in the construction of concepts/categories and provides analytical and epistemological tools for the paradigmatic ordering of concepts/categories.

### Study Site

The data was collected at a federal university hospital in the city of Rio de Janeiro - RJ. The study involved the medical and surgical clinic sectors, as it was considered that these places produce a significant amount of waste from direct and indirect nursing care for patients.

### Selection Criteria

Nurses and nursing technicians took part in the study. Inclusion criteria were: having at least one year’s professional experience in the institution, in the research setting in question, and in direct patient care as a nurse or nursing technician. Participants who were away from work, on leave or on vacation were excluded. Participants were invited in person, in the study setting, forming a convenience sample. No participants refused or dropped out.

### Sample Definition

The sample was defined by theoretical saturation, as determined by the method, when the researchers find that the categories and their respective subcategories have reached sufficient theoretical density to jointly explain the object of study. In GT, the process of theoretical saturation is favored because the data collection takes place in parallel with the analysis, as the researchers analyze the data after each interview and then return to the research site^(12,13)^. Thus, 32 professionals took part in the research.

### Data Collection

Data collection took place between January and August 2022, using semi-structured interviews. The interviews took place in individual meetings, in a private environment, inside the study setting, at previously agreed times that did not compromise the interviewees’ work activities. These interviews were digitally recorded (audio) and lasted an average of 30 minutes each, and were not repeated with the same participant. Because the method seeks to capture the meanings that emerge during the interviews^(13)^, the interviews were not returned to the participants for adjustments/additions.

The researcher responsible for data collection, who is a nurse, has developed skills for the methodological approach described in this study with the research group to which she is affiliated, as well as in her experiences as a researcher in other similar scientific investigations. There were no personal or professional conflicts of interest in carrying out the study in the chosen setting or with the research participants involved.

### Data Analysis and Treatment

For this research, we adopted the constructivist perspective of the Corbinian school of GT, which, in common with the others, has a comparative approach, constant questions, theoretical sampling, elaboration and integration of concepts^(12,13)^. However, it differs in its paradigmatic model, which is made up of three dimensions: conditions, actions-interactions and consequences.

The dataset from the interviews was analyzed following the coding stages of the GT, from the Corbinian school, which are: open, axial and selective integration. In open coding, the data was segmented into distinct parts, rigorously examined and compared in search of similarities and differences^(13)^. At this stage, the initial codes are provisional.

The interviews were transcribed in Microsoft Office Word® 2016, and their data were imported into NVIVO® 12. This software helps the researcher in the process of coding the texts and grouping the information for conducting studies with GT. It should be noted, however, that the simultaneous analytical process (data collection and analysis) was maintained even with the use of the aforementioned software. Initially, preliminary codes were formed, based on the identification of specific properties and dimensions of the transcribed interviews. The preliminary codes are more descriptive of the data. After gathering preliminary codes from multiple interviews, the process of grouping these codes by similarities and differences was intensified with the help of NVIVO® 12.

The grouping of preliminary codes by similarities and differences gave rise to conceptual codes, which are presented as an abstract representation of a fact, object or action that the researcher perceives as significant in the data^(13)^. The grouping of conceptual codes by similarities gave rise to categories. Once the category has been identified, the researcher can begin to develop it into specific properties and dimensions. Properties are general or specific characteristics or attributes that outline, define and give meaning to a given category^(13)^.

One of the characteristics of GT consists of comparative analysis in all the analytical phases. Thus, not every concept (connections of similar conceptual codes) is dense enough to form a category, but it can add meaning around one and become a subcategory. In this way, axial coding begins, which allows the researcher to relate categories to related subcategories. Axial coding therefore takes place around the axis of a category, associating categories at the level of properties and dimensions, to add depth and structure^(13)^.

To make sense of the categories, GT uses the paradigmatic model as an analytical tool. This model consists of the following resources: conditions, which are the reasons given by the informants for the occurrence of a given fact, as well as explanations of why they respond in a given way to an action; actions-interactions: response expressed by the participants to events or problem situations; consequences: refer to the expected or actual results of actions and interactions^(11)^.

Integration is the last stage in the construction of the GT, and represents the process of refining and integrating categories in order to reveal the central category. In integration, the categories are organized around a central explanatory concept, which begins with the first steps of the analysis and usually doesn’t end until the final draft^(13)^.

Concomitant with the process of coding the data, and just as important, is to direct the analysis in terms of context and process. This analysis was recorded in memos written and organized in NVIVO® 12 by the main researcher.

### Ethical Aspects

The research project for this investigation was cleared on August 30, 2021 by the Human Research Ethics Committee under opinion number 4.941.218. It met the requirements of Resolutions 466/12 and 580/18 of the National Health Council. After giving their consent, the participants signed an Informed Consent Form. To ensure anonymity and confidentiality, the interviewees are designated in their interviews (excerpts) presented throughout the article as follows: EN (nurse) and TE (nursing technicians), followed by the number of their respective interviews.

## RESULTS

Ten nurses and 22 nursing technicians took part in the study. Of the nurses, 9 were specialists. The average length of training was 12 years and 9 months, while the average length of professional experience in the research setting was five years and 10 months.

The data presented in this article make up the dimension of strategic actions and interactions, signaled in the paradigmatic model of the GT, whose category/concept constructed was entitled *Sustainable Nursing Care and its Connections with Healthcare Waste Management*, and is based on four subcategories that support the aforementioned concept based on the complexity involved in the competencies that make up sustainable care, from the development of an ecological awareness, to the practical consequences for the use and rational disposal of HCW. The first subcategory is presented below.

### Strategies for Managing Healthcare Waste in Hospitals

The appropriation of the concept of sustainability as a mediator in planning nursing care permeates strategies that can be adopted in the hospital context to manage HCW. However, social and environmental responsibility in relation to the use of hospital resources must be understood, starting with individual actions, such as rationalizing materials that can contribute to a more sustainable institution. This reality is glimpsed by the participants.

(...) *calculate the material you’re going to use* (...) *use less material, if it’s not necessary, don’t use the material. For example, not taking that large amount of material that won’t be used at that moment, which we often end up taking and won’t use, I think we can save a bit too.* (TE 01)

The results show that a conscious and sustainable approach to the use of material resources, with an emphasis on carefully calculating the quantity needed to avoid waste, can lead to a reduction in the generation of HCW and, consequently, economic and environmental impacts, as shown below:

*If we could get everyone to understand that the rational use of materials can help the hospital and society, perhaps things would change. I say the hospital because of the economy, and society would also benefit from the protected ecology.”* (EN 09)

For nursing professionals, the lack of control over the use of materials and the need for a professional responsible for auditing and accounting for the materials used, with the aim of avoiding and/or reducing waste and ensuring that there are sufficient supplies to meet the institution’s needs, may bring up problems such as: lack of essential materials, overspending and waste of resources. Implementing measures to monitor and control the use of materials can help reduce these impasses and ensure more efficient and sustainable use of resources, as the participants point out.

*I believe that there is a lack of greater control over what we use, which is why I think there is often a lack of material,* (...) *there would have to be someone to audit this, it’s not just us writing down what we’ve used* (...) *there would have to be someone to audit it, to account for how many are being used.*

*There needs to be someone to supervise this issue better, to help control the excessive use of materials, so that we and the patient don’t run out*. (TE 21)

The data shows that there is no consensus on the importance of knowledge in nursing about managing resources and spending on materials that have an impact on the production of HCW.

*Here, as we are in a somewhat senior position, it’s part of management. I need to know that what I have will last for so long, right? What I spend per week*.” (EN 01)

*I think everyone knows the importance of not wasting, not throwing material where it doesn’t belong. It’s something we’ve known since we started working here, we don’t need a course on it*. (TE 19)

For nursing professionals, HCW is not only affected by activities related to materials resulting from direct patient care. In this sense, they point out the group of electronic waste, such as batteries, while recognizing the importance of proper final disposal, given the ecological impact that this waste can have.

(...) *we collect these batteries there so that we don’t dispose of them inappropriately, and also with the aim of exchanging them. We have hundreds (lots) of used batteries to exchange.”* (EN 08)

The complexity imbued in the meanings that nursing professionals attribute to HCW in the hospital context is directly related to the interactions they perceive between this reality and the actors involved in it. In this sense, they attribute importance to hospital hygiene professionals (general services), while at the same time perceiving as worrying the devaluation of the reality in which these professionals are neglected in qualification and training programs for the management of HCW, as exemplified below:

(...) *the category that is looked down on the least, it’s like looking at an ant, that’s how cleaners are seen, you know?* (...) *they also influence this issue of health waste*. (TE 13)

When it comes to technical and structural issues that are easy to resolve, the nursing professionals considered it important to improve the conditions of resources for correct disposal by improving the identification of collection containers. By way of example, the following excerpts highlight the importance of properly identifying the different types of bins in a hospital.

*Some garbage cans are only identified at the bottom, but I think there should be better identification, even on the lid, so that people can see that it’s only infectious waste*. (EN 08)

(...) *if we could improve the structure of the garbage cans, the identification of the garbage cans, really identify them, not just put them there, but say, describe the waste that should be there.* (TE 19)

According to the nursing professionals surveyed, clearer and more visible identification of the collection containers is essential so that everyone can easily identify what type of waste the collector is for, so that the waste disposed of can be properly segregated and reduce the chance of cross-contamination or other problems associated with inadequate hospital waste management. The data also revealed the importance of interactions in the field of professional knowledge for the management of HCW, as highlighted in the following subcategory.

### Permanent Education and the Promotion of Awareness-Raising Actions and Environmental Education in the Hospital Context

This subcategory reveals the lack of training and debates on the subject of “HCW management” and the need to strengthen continuing education aimed at promoting care with a view to sustainability in health institutions. One of the solutions to reducing the waste of materials and the handling of HCW in the hospital context would be through contextualized training in permanent education for all the professionals involved.

*We need to learn more about the importance of all this, not just how to do it, but also why we dispose of it in a certain way. That’s why training like this would be better, something that would allow us to take a broad view.* (EN 04)

The results highlight the need for ongoing education, not only in relation to patient management and care, which is usually widespread and necessary, but also in relation to the correct disposal of waste, such as batteries and sharps. The observation that a specific course on correct disposal was never offered indicates the lack of attention paid to the issue and the need to include this topic in the training offered to health professionals.

(...) *here, I think it’s interesting the training we have, from time to time, we have training on how to manipulate and handle patients, but there has never been a course on the correct disposal of sharps. The head of the sector does it, as well as batteries.* (TE 02)

*So, I think that if we spread the word like this, with posters and information and also training, which we can do in the morning, sometimes even in two sessions, it’s a quick thing. The staff has a good response, all the sectors, the staff does it properly, you know? So, I think it’s a good open door to set something like this up and raise awareness among nursing staff*. (EN 01)

*We need to publicize this subject more, make it more common in our daily lives, because it’s all the time, every day on duty, wherever you are, there’s often incorrect disposal, because there’s only that garbage can and nobody cares.* (EN 04)

The importance of awareness and training to improve professional practice was highlighted. For the participants, awareness of the correct disposal of materials is fostered through educational processes. For them, training needs to be carried out regularly to maintain awareness and good results.

*This awareness only comes with training, and training needs to be done every 3 months, because people forget. We did a training course on venipuncture, venous access, infection and it was a success, they loved it, the numbers improved. I don’t know what, but after a while, cases started again, the training needs to be constant, it really needs to be cyclical.* (EN 08)

### Supervision: the others’ view of Healthcare Waste Management Actions

Nurses and nursing technicians, due to the lack of environmental awareness among health professionals, need to be supervised by a responsible professional for the management of HCW in the hospital context, which can happen through periodic visits to the sectors, with the aim of guiding/training the health professionals. In addition, the participants pointed to cross-cutting contexts in which such approaches can take place, including the Hospital Infection Control Commission (HICC), which they mentioned.

*The HICC has a nurse who comes in a couple of times a week and does rounds of the ward. If she finds a needle, she says: ‘Be careful, the needle has to be in the collector!’ (...) ‘Don’t mix this with that!’, she goes through and gives a quick light, especially, not just on the sharpened objects part, but if she finds that medication open, she says* (...) *she always gives guidance*. (TE 12)

The participants recognize that the actions and interactions established with the HICC nurse, based on her guidance, are important for maintaining patient safety and preventing health-related infections. In addition, their approaches offer objective redirections that help them in their day-to-day decision-making regarding the management of HCW. The actions-interactions for better HCW management are not limited to healthcare professionals, but also reach hospital hygiene professionals, whose meanings are centered on the co-responsibility of supervision.

*Once, I accidentally threw a piece of equipment in the wrong place, and the cleaning guy said: ‘You can’t throw it here! So I became more aware of other opportunities*. (TE 04)

The data corroborates the systemic perspective that strategies for managing HCW, in the hospital context, assume a non-linear logic between the parties involved, in which all members are important. At the same time, the following subcategory highlights nursing as strategic for balancing this reality.

### Nursing as a Chaotic Attractor for Positive Change in hcw Management

The concept of “nursing as a chaotic attractor” emerges from this subcategory and refers to nursing’s ability to be an agent of change in the context of HCW management, as it uses its skills to promote, through sustainable care, practices that are more conscious and safer for the environment and for the health of other professionals and patients.

From the point of view of participants, nursing can bring about the necessary change in the correct management of HCW, and this ability stems from stimulating a change in thinking that can modify behavior towards sustainable practices. From this reality, there are better possibilities for implementing more effective waste management strategies in the field of education and raising awareness among health professionals about environmental issues, as well as the search for innovative and collaborative solutions to improve the management of HCW. Thus, the data shows that nursing can play a fundamental role in promoting more sustainable practices and preventing environmental and health damage resulting from the management of HCW.

*Nursing can transform reality, it has that power*. (EN 07)

*Nursing is always willing to learn, we find a way to do everything, I think we should think of a better way of working to optimize our work, right?* (TE 02).

## DISCUSSION

The systemic conception of complex phenomena signals the need for thinking capable of establishing connections between the parts, in a logic that goes beyond the simplistic relationship of cause and effect^(14,15)^. It is in this sense that the inappropriate production of waste can result in serious consequences for the maintenance of the ecosystem, with rapid degradation of the environment associated with global warming, so as to determine climate change. Therefore, it is appropriate to corroborate the need to reform thinking in order to think about reform^(5)^, since changing thinking that results in changing behavior is a critical challenge for the global community in terms of preserving life^(15)^.

Despite the above, according to Complexity Theory, changes in thoughts and actions do not occur in a linear fashion. In this respect, we have what Morin characterizes as the ecology of action, namely: when a phenomenon undergoes a set of connections, it tends to start moving in a way that its consequences are no longer known, due to a lack of control over the nature of its interactions^(5)^. Consequently, the management of HSR must be achieved through a complex perspective capable of connecting patient care to ecological care, since the complex means what is woven together^(5,15)^. Therefore, there is a need to understand the connections between the parts, which together form the whole, providing a systemic view of reality^(5)^.

Nursing care with a view to sustainability, based on a well-designed action plan, may be able to reduce the amount of HCW without reducing the quality of care offered to patients. Nursing professionals therefore need to be aware of their critical role in effective waste management, as indicated by the data in this study, because they are the ones who, in most of the procedures carried out, separate the waste at the point of generation, for example. In this context, efforts to identify and eliminate unnecessary sources of waste generation can have a positive impact on the effectiveness of developing a sustainable ecosystem, which involves the health sector^(16)^.

In the production of health and nursing care, depending on the interventions carried out, it is inevitable that waste will be generated. However, thinking about care in a sustainable way, without wasting materials and disposing of waste correctly, should be part of the daily lives of health professionals^(17)^, given the importance of managing HCW being conceived and processed from a systemic perspective, connected between all the parties, which makes this phenomenon a responsibility of health workers, as well as decision-makers in the legislative and public management spheres^(18)^. Furthermore, this perspective goes back to what Morin categorizes as the principle of self-organization, which is anchored in two concepts: autonomy and dependence. The relationship between these concepts and sustainable care and the management of HCW highlights the fact that nurses’ autonomy in making decisions aimed at providing sustainable care also depends on the environmental culture in which they live. For this reason, and based on complexity, health workers are understood as eco-self-organizing beings, as they depend on their culture and ecological awareness to exercise their autonomy in the context of HCW management.

In addition to other agendas, rethinking consumption patterns and reducing waste generation, ensuring that they are sustainable, is a call to all nations for sustainable development. With this in mind, the UN’s 2030 Agenda, through the SDGs, aims to protect the planet from degradation, above all through sustainable consumption and production, through sustainable management of natural resources, as well as by taking urgent action on climate change. These actions aim to ensure that the biosphere can support the needs of present and future generations^(11,18)^.

In this context, there is room to discuss reverse logistics, which is the process of managing products after final consumption, with the aim of reducing environmental impact and promoting sustainability. Thus, reverse logistics is the management of waste generated by products and packaging after use, with a view to recycling, reuse or appropriate final disposal^(19,20)^.

In view of the connections related to the subject, the management of HCW is a complex phenomenon because it also involves the multidimensionality of the actors involved in its process. The shared responsibility of manufacturers, consumers and governments in waste management should therefore be highlighted. Manufacturers are responsible for implementing recycling systems and producing more sustainable products; consumers have the role of separating and disposing of waste properly; and governments must establish public policies that encourage reverse logistics and regulate waste management; direct patient care professionals have the responsibility to act, supervise and demand sustainable actions^(20)^. This reflection is important because it highlights proper waste management in a hospital, including the need to ensure that people can easily identify the type of waste they should dispose of, as demonstrated by the nurses in this research. Thus, by improving the identification of waste containers, it is possible to increase the effectiveness of hospital waste management and reduce the risk of problems associated with contamination or other public health hazards^(21,22)^.

In order to implement HCW management safely, it is necessary to use procedures, standards and routines at the institutional level. To this end, professionals must be trained and encouraged to take actions that minimize the generation of HCW, with management based on accessible and resolutive methods. It is important to emphasize that, in addition to all this, it should also be considered that reducing the volume of hospital waste is only possible when the activities of the sectors responsible for generating this waste are integrated into daily work, along with the awareness of all the professionals involved in the management of HCW^(22)^.

Finally, environmental assessments are necessary to understand the wide-ranging impact of HCW, both on public health directly and on the environment. From this perspective, it should be noted that hospitals are also seen as institutions that consume a high volume of disposable plastic products to minimize infections, as well as different medicines and medical supplies. Therefore, detailed instructions or manuals on waste handling should be provided to healthcare professionals for proper separation and disposal to reduce the volume of waste generated^(23,24)^.

According to the data, continuing education is a fundamental strategy for promoting environmental education actions in the hospital environment, with a view to the proper management of HCW. Some strategies that can be adopted include regular training for health professionals on the correct procedures for segregating and disposing of waste, encouraging the use of more sustainable materials and equipment with less environmental impact, awareness campaigns for patients and their families on the importance of proper waste management, among others^(24)^. Furthermore, in relation to healthcare professionals, this reality is corroborated by the results found in a study in which the majority of those surveyed revealed that they had not received any training/education related to waste management (56.8%)^(25)^. However, it should be emphasized that, for the sake of complexity, the educational process must break the pathology of knowledge, i.e. isolated, decontextualized knowledge^(5)^. Thus, education for the transformation of positive practices related to HCW management must involve a multidimensional perspective and, consequently, ecology as an inseparable part of health and nursing.

Intervention measures are important to fill the gaps in knowledge. In addition, practices and attitudes should be supported by training in HCW management for waste handlers and managers, in order to foster greater change in the practice of healthcare professionals^(26)^. In this sense, education on the management of HCW is a necessity for sustainable processes related to healthcare, in which all members of the hospital should be encouraged to participate in education on the value of managing this waste, the aim of which is to achieve the most sustainable environment possible^(27)^.

By highlighting the importance of supervision, the research data reinforces that this is an important practice in the management of HCW, as it allows the professionals involved in the activities to take a critical and reflective look at their actions. Supervision also allows professionals to receive feedback on their work and identify possible failures or opportunities for improvement. It also promotes the sharing of knowledge and experiences between professionals, which can contribute to improving waste management in the hospital context^(28)^.

The role of nursing in the HICC is relevant, especially as it looks for ways to learn more about infections in the healthcare unit, as well as carrying out permanent education actions that include the entire nursing team and other members of the healthcare team. The importance of these bodies in terms of prevention stands out, since they assess the risks present in the workplace, carry out preventive actions, as well as guiding workers on issues related to health and safety^(29)^.

Non-compliance with the guidelines regulating the management of HCW may be due to a lack of supervision by the authorities and the absence of strict rules and procedures. Therefore, it is important to emphasize the importance of drawing up rules and regulations followed by educational enforcement, as well as regular monitoring to reinforce the importance of adherence and ensure compliance with the protocols related to HCW management^(24,26)^. This understanding is in line with the principle of the ecology of action, in which an action does not depend solely on the will of the person practicing it, but mainly on the contexts in which it takes place, the social, biological, cultural and political conditions that drive it and feed back into its development or disappearance^(5)^.

Therefore, when considering the importance of HCW management as a complex phenomenon, rooted in multiple factors that have a potential impact on people’s quality of life and on the environment, it is necessary to define a starting point for an investigation capable of understanding this phenomenon based on human action in everyday healthcare work^(8)^. Because it is a complex phenomenon, it is necessary to demarcate the context of its emergence and development^(5,14)^, which, in this problematization, is centered on the hospital. However, the understanding of context is not limited to the physical structure in which a given phenomenon is positioned, but also involves the people involved in the social interactions that influence its development. The projection of this research focuses on nursing.

Due to the unique nature of the phenomenon under study, direct replication of the research results can be challenging. Replicating the same results in different settings may present specificities that signal the study’s limitation, namely: the need to reach other sample groups, such as other members of the healthcare team and the cleaning staff. The setting of the study may also have limiting implications, since it took place in a university hospital which may be subject to specific educational actions. Therefore, similar studies in private or public hospitals other than university hospitals may reveal different results to this study.

## CONCLUSION

The complexity involved in the actions and interactions that permeate nursing professionals’ meanings about the management of HCW is directly related to the perspective of sustainable care. These connections are established as these professionals recognize and value the understanding of social and environmental responsibility for the use of material resources, with due concern for the rational use of these resources and reduction of waste that affects waste generation and impacts the institutional economy and, consequently, the health sector. In this sense, the participants pondered the relationship between the rational use of materials, waste generation, the health economy and the quality of care. From these connections, the complex meaning of sustainable care emerges.

The results of the survey showed meanings related to the context of organizational culture and work processes related to the management of HCW, from actions that promote the correct classification of waste, training and capacity building for the professionals involved in HCW management; to the monitoring and control of the waste generated, from its generation to its final disposal, in order to guarantee environmental and public health safety; among others.

The complexity paradigm therefore establishes important links with the way that nursing learns about the sustainability required for ecological care capable of thinking/doing HCW management based on the relationship between context, environment, individuals and ecology.
